# Overexpression of Cyclooxygenase-2 in Malignant Peripheral Nerve Sheath Tumor and Selective Cyclooxygenase-2 Inhibitor-Induced Apoptosis by Activating Caspases in Human Malignant Peripheral Nerve Sheath Tumor Cells

**DOI:** 10.1371/journal.pone.0088035

**Published:** 2014-02-06

**Authors:** Michiyuki Hakozaki, Takahiro Tajino, Shinichi Konno, Shinichi Kikuchi, Hitoshi Yamada, Michiro Yanagisawa, Jun Nishida, Hiroyuki Nagasawa, Takashi Tsuchiya, Akira Ogose, Masafumi Abe, Hiroshi Hojo

**Affiliations:** 1 Department of Orthopaedic Surgery, Fukushima Medical University School of Medicine, Fukushima, Japan; 2 Department of Pathology and Diagnostic Pathology, Fukushima Medical University School of Medicine, Fukushima, Japan; 3 Department of Orthopaedic Surgery, Hirosaki University Graduate School of Medicine, Aomori, Japan; 4 Department of Orthopaedic Surgery, School of Medicine, Iwate Medical University, Iwate, Japan; 5 Department of Orthopedic Surgery, Akita University Graduate School of Medicine, Akita, Japan; 6 Department of Orthopaedic Surgery, Yamagata University Faculty of Medicine, Yamagata, Japan; 7 Division of Orthopedic Surgery, Department of Regenerative and Transplant Medicine, Niigata University Graduate School of Medical and Dental Sciences, Niigata, Japan; 8 Tohoku Musculoskeletal Tumor Society, Yamagata, Japan; University Hospital of Navarra, Spain

## Abstract

**Background:**

Cyclooxygenase-2 (COX-2) is a key enzyme in the conversion of arachidonic acid to prostanoids, and its activation is associated with carcinogenesis as well as inflammation. The antitumor effect of selective COX-2 inhibitors has been noted in various malignancies. Malignant peripheral nerve sheath tumor (MPNST) is a rare and aggressive soft tissue sarcoma for which effective treatments have not yet been established. The purpose of this study was to investigate a potential therapeutic role of COX-2 in MPNST.

**Methods:**

We evaluated the expression of COX-2 in 44 cases of high-grade MPNST using immunohistochemical staining and compared the staining results with the characteristics and outcome of the patients. We also investigated the antitumor effect of etodolac, a selective COX-2 inhibitor, on MPNST cells *in vitro* using the MPNST cell line, FMS-1.

**Results:**

Overexpression of COX-2 (≥50% positive cells) was observed in 29 cases (65.9%), was significantly associated with a poor overall survival (P = 0.0495), and was considered an independent risk factor for a poor outcome by the results of both univariate and multivariate analysis. Etodolac induced apoptosis of FMS-1 cells through the activation of caspase-8, -9, and -3. Moreover, several caspase inhibitors significantly inhibited etodolac-induced apoptosis.

**Conclusions:**

Selective COX-2 inhibitors including etodolac had an antitumor effect on MPNST cells, and their use holds promise as a novel therapeutic strategy for patients with MPNST to improve their prognoses.

## Introduction

Malignant peripheral nerve sheath tumor (MPNST), also called malignant schwannoma or neurofibrosarcoma, is a rare soft tissue sarcoma, accounting for approximately 5% of soft tissue sarcomas. Approximately half of MPNSTs manifest in patients with neurofibromatosis type 1 (NF1; von Recklinghausen disease) [1], and patients with NF1 have a 5–10% lifetime risk of MPNST [2], [3]. MPNST frequently shows highly aggressive behavior, resistance to multi-agent chemotherapy and radiation therapy, and fatal metastasis. About 60% of patients with MPNST die of this disease, and the overall 5- and 10-year survival rates are 34% and 23%, respectively [1]. New therapeutic developments including molecular-targeting drugs based on molecular genetic and biological characterizations of MPNST are required to improve the aggressive course and fatal prognosis of this disease.

Cyclooxygenase (COX), also known as prostaglandin H2 synthase or prostaglandin endoperoxide synthase, is a key enzyme in the conversion of arachidonic acid to prostanoids [4]. COX-2 is one of two COX types, the other being COX-1. COX-2 is undetectable in most normal tissues, but it can be induced in various cell types by pro-inflammatory agents, growth factors, and carcinogens [5]. Overexpression of COX-2 and its association with worse prognosis in various malignancies, especially in bone and soft tissue sarcomas [6]–[10], has been reported. COX-2 activation leads to the enhancement of cell proliferation and migration, suppression of apoptosis, stimulation of neovascularization, and alteration of intercellular adhesion, all of which are involved in carcinogenesis [11]. There have been several reports on the antitumor effects of some selective COX-2 inhibitors for bone and soft tissue sarcoma cells, including the induction of apoptosis [12]–[21]. However, overexpression of COX-2 in human MPNST and the antitumor effect of the selective COX-2 inhibitors on the growth of human MPNST cells have not been analyzed in detail.

In this study, we examined the expression of the COX-2 protein in human high-grade MPNST specimens by immunohistochemical techniques and analyzed the relationship between COX-2 overexpression and prognosis. In addition, we examined the antitumor effect of inducing apoptosis through caspase activation by a selective COX-2 inhibitor, etodolac, on a human MPNST cell line *in vitro*.

## Results

### Association of COX-2 Overexpression with Clinical and Pathological Features

**Figure 1:**
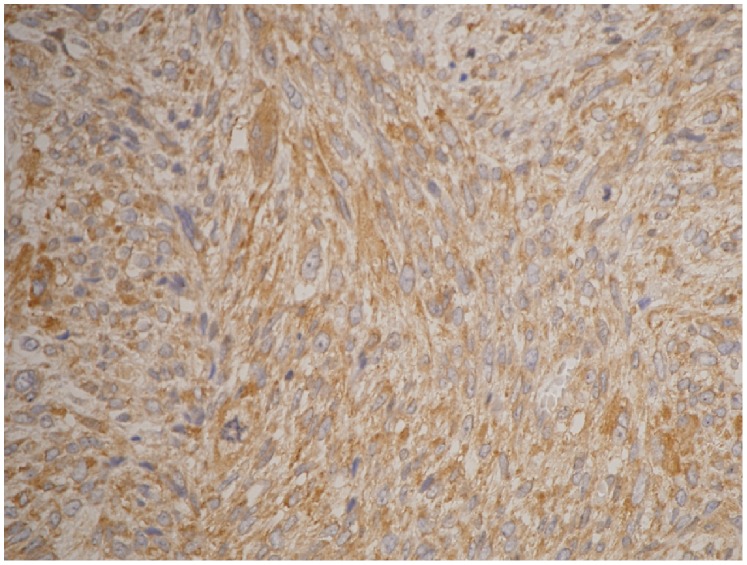
Diffuse, strong, and cytoplasmic-positive immunoreactivity for COX-2 in MPNST (immunohistochemical score, 4+) (immune-peroxidase stain, ×400).

The results are summarized in Table 1. COX-2 was expressed in 41 (93.2%) patients, and COX-2 overexpression (immunohistochemical score 3+ and 4+) was observed in 29 (65.9%) of the 44 patients based on the immunohistochemical score: 0, 3 cases; 1+, 2 cases; 2+, 10 cases; 3+, 15 cases; and 4+ in the 14 cases. COX-2 staining in the MPNSTs was consistently observed in the tumor cells with a strong and cytoplasmic pattern (Figure 1).

**Table 1:** Statistical relationship between immunohistochemical result of COX-2 and other variables.

Variable	No. of patients	COX-2 overexpression			P-value
		Negative	Positive	%*	
Age (y)					
<50	20	10	10	50.0	0.04
≥50	24	5	19	79.2	
Sex					
Male	21	9	12	57.1	0.24
Female	23	6	17	73.9	
NF-1					
Absent	23	4	19	82.6	0.01
Present	21	11	10	47.6	
Relationship to peripheral nerve					
Absent	20	9	11	55.0	0.16
Present	24	6	18	75.0	
Location					
Trunk	20	5	15	75.0	0.25
Extremity	24	10	14	58.3	
Depth					
Deep-seated	31	9	22	71.0	0.43 (excluding NA)
Superficial	12	5	7	58.3	
NA	1	1	0	0	
Size (cm)					
<10	22	8	14	63.6	0.75 (excluding NA)
≥10	19	6	13	68.4	
NA	3	1	2	66.7	
Distant metastasis at time of diagnosis					
Absent	33	12	21	63.6	0.58
Present	11	3	8	72.7	

*Percentage of positive cases; NA, not available.

Statistical analysis indicated that COX-2 overexpression was significantly associated with the patient age (<50 versus ≥50 years) (P = 0.04) and background of NF1 (P = 0.01) (Table 1). There were no significant differences between COX-2 overexpression and the other variables, including sex, anatomical site (trunk versus limb), depth (deep-seated versus superficial), size (<10 versus ≥10 cm), association with peripheral nerve, and distant metastasis at time of diagnosis (Table 1).

**Figure 2:**
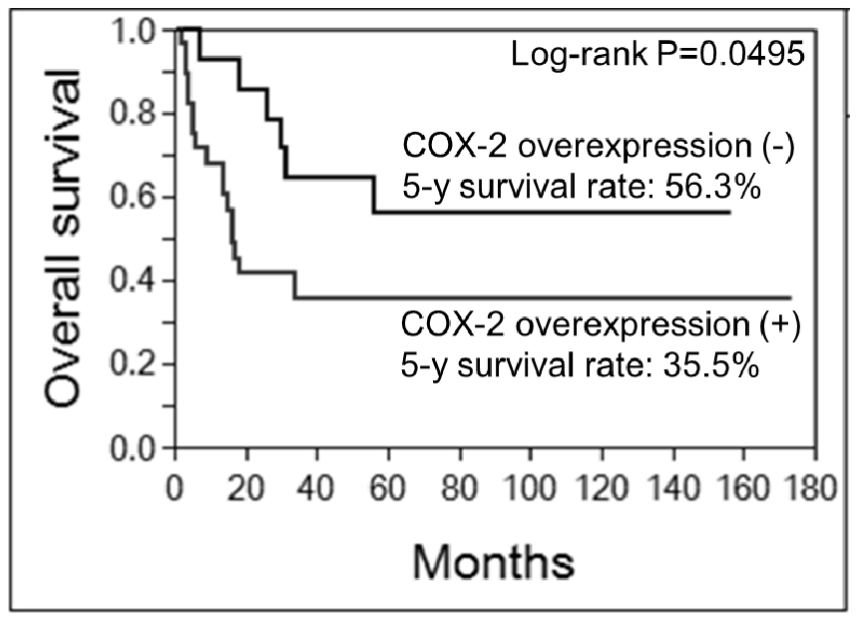
Kaplan-Meier curve of overall survival evaluated for COX-2 overexpression. Overexpression of COX-2 was significantly associated with a decreased probability of overall survival (P = 0.0495).

Log-rank analysis revealed that COX-2 overexpression was significantly associated with a decreased probability of overall survival (P = 0.0495) (Table 2 and Figure 2). The probability of survival at 5 years was 35.5% or 56.3% in patients with or without COX-2 overexpression, respectively (Table 2 and Figure 2). Each of the following was also associated with poor prognosis from the result of log-rank analysis: the location in deep tissue, large tumor size (≥10 cm), the presence of distant metastasis at the time of diagnosis, non-curative surgical treatment, and additional treatment with chemotherapy (Table 2).

**Table 2:** Log-rank analysis of overall survival.

Variable	No. of patients	5-y survival rate (%)	P-value (Log-rank test)
Age (y)			
<50	20	40.4	0.78
≥50	24	43.2	
Sex			
Male	21	25.3	0.11
Female	23	56.3	
NF-1			
Absent	23	39.9	0.70
Present	21	41.0	
Relationship to peripheral nerve			
Absent	24	46.1	0.48
Present	20	35.5	
Location			
Trunk	20	26.2	0.37
Extremity	24	49.7	
Depth			
Deep-seated	31	32.2	0.09 (excluding NA)
Superficial	12	66.7	
NA	1		
Size (cm)			
<10	22	64.3	0.009 (excluding NA)
≥10	19	22.6	
NA	3		
Distant metastasis at time of diagnosis			
Absent	33	54.5	<0.0001
Present	11	0	
Surgical treatment			
Curative	24	66.1	0.0002
Not curative*	20	9.8	
Chemotherapy			
Yes	11	10.4	0.0434
No	33	53.8	
Radiation therapy			
Yes	14	23.8	0.38
No	30	48.7	
COX-2 overexpression			
Negative	15	56.3	0.0495
Positive	29	35.5	

*including the cases in which only a biopsy was performed; NA, not available.

Several variables were tested to assess whether they affected overall survival. The univariate and multivariate analyses with the Cox proportional hazards model revealed that large tumor size (≥10 cm), the presence of distant metastasis at the time of diagnosis, non-curative surgical treatment, and COX-2 overexpression were independent risk factors for poor outcome (Table 3). The location in deep tissue and treatment with chemotherapy were not significantly associated with poor prognosis by univariate or multivariate analysis (data not shown).

**Table 3:** Univariate and multivariate analysis of overall survival (Cox proportional hazards model).

Variable	Univariate analysis			Multivariate analysis		
	Hazard ratio	95% CI	P-value	Hazard ratio	95% CI	P-value
Size (cm)						
<10	1	1.29–8.23	0.011	1	1.09–7.43	0.0318
≥10	3.11			2.72		
Distant metastasis at time of diagnosis						
Absent	1	2.83–18.58	<0.0001	1	1.81–17.82	0.0029
Present	7.20			5.56		
Surgical treatment						
Curative	1	1.90–11.09	0.0005	1	1.33–11.40	0.0124
Not curative*	4.42			3.75		
COX-2 overexpression						
Negative	1	1.02–6.99	0.0456	1	1.05–10.23	0.0400
Positive	2.49			3.04		

*including the cases in which only a biopsy was performed; CI, confidence interval.

### Etodolac-induced Cell Death *in vitro*

**Figure 3:**
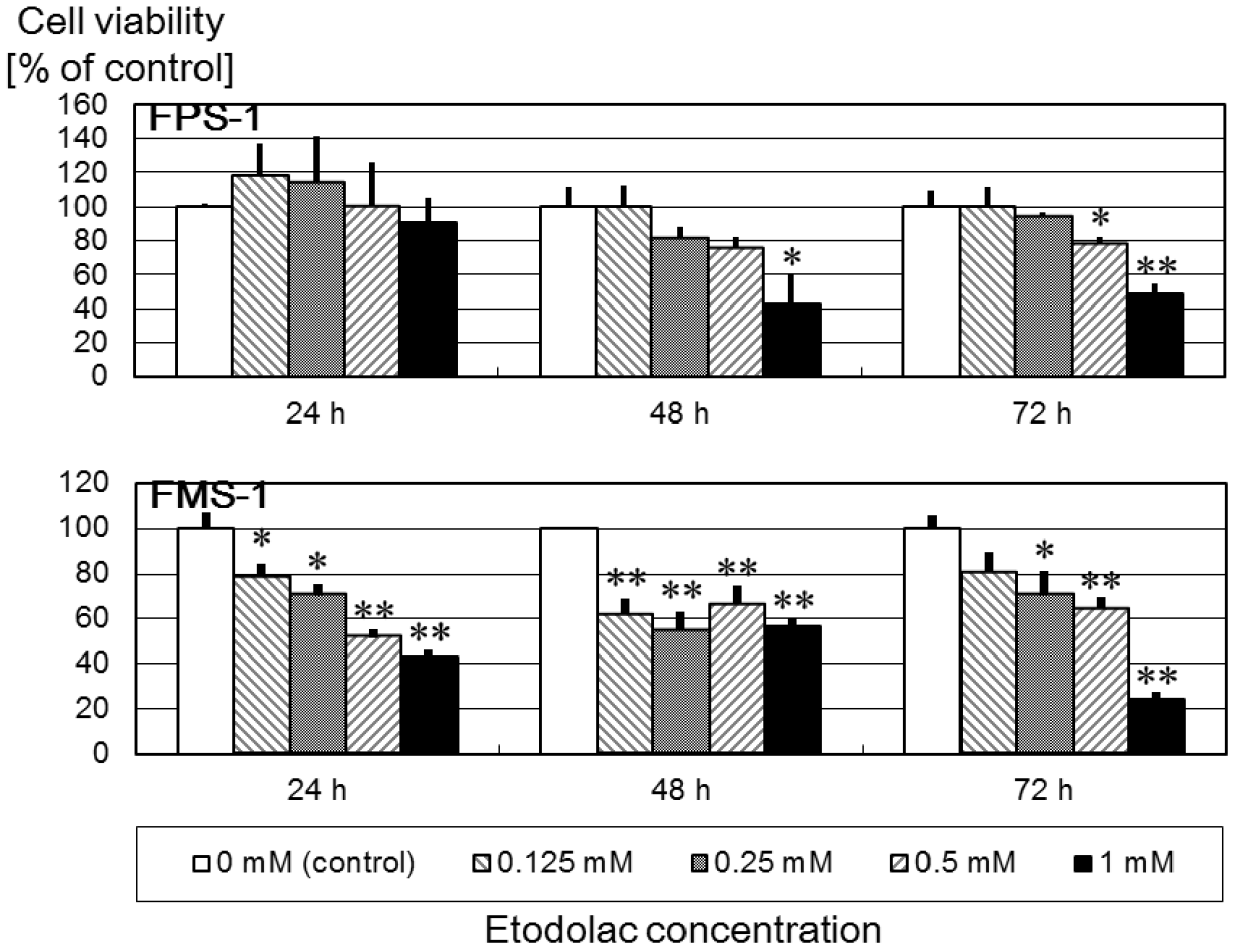
Effect of etodolac treatment on FPS-1 and FMS-1 cells (n = 5). Etodolac significantly reduced cell viability of FMS-1 cells in a dose-dependent manner. P-values compared with the control were calculated based on Student’s t-test (*, P<0.05; **, P<0.01).

In the undifferentiated pleomorphic sarcoma (UPS) cell line, FPS-1 [22], cell viability was significantly inhibited at only higher concentration and longer period. Conversely, the MPNST cell line, FMS-1 [23], cell growth was inhibited in a dose-dependent manner, and cell viability was significantly inhibited at all times (Figure 3).

### Morphological Analysis and DNA Fragmentation Analysis of Dead Cells

**Figure 4:**
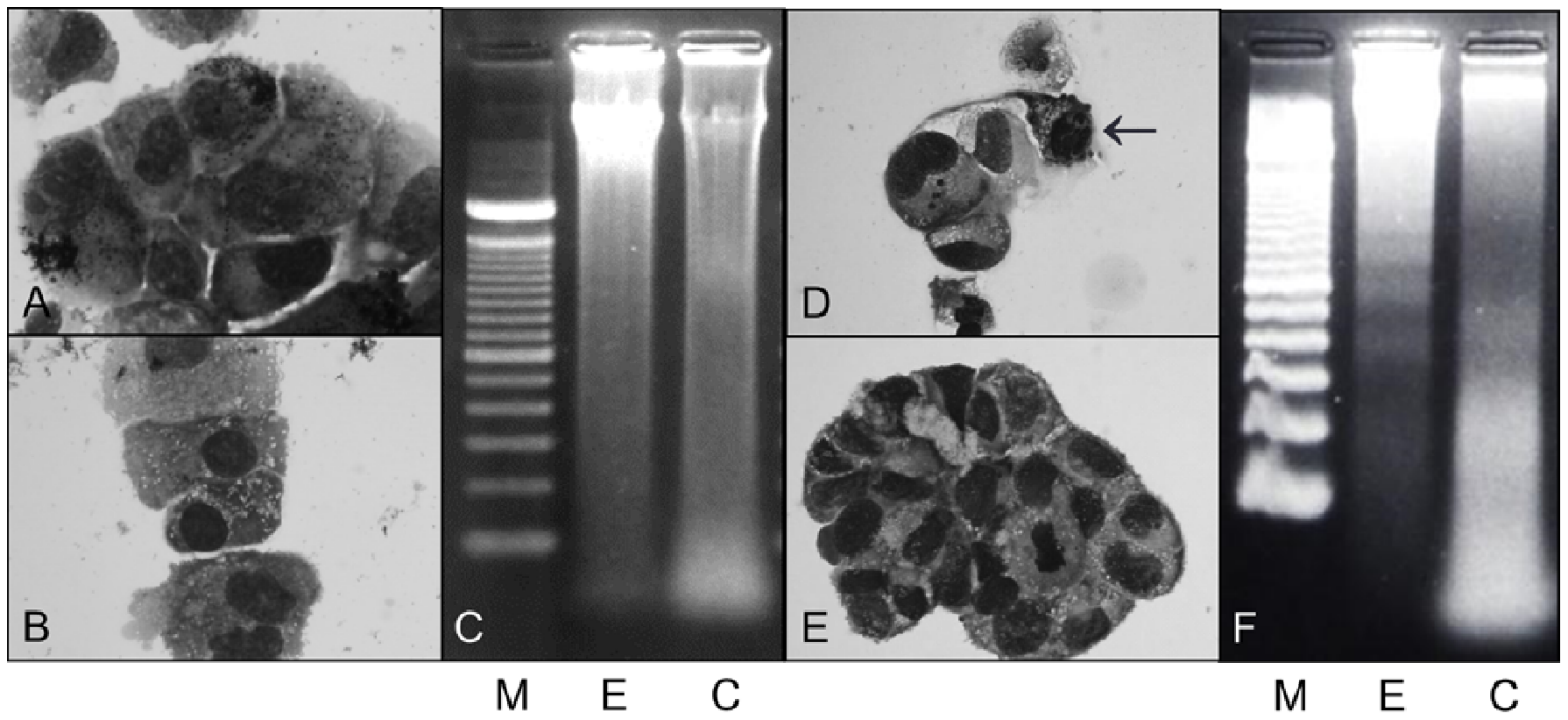
Morphology of FPS-1 (A) and FMS-1 (D) cells after etodolac treatment for 72 h. Nuclear fragmentations were observed only in FMS-1 cells (D, arrow). Both in FPS-1 (B) and FMS-1 (E) without etodolac treatment, cells were viable and no apoptotic cells were observed (May-Giemsa staining, ×400). Evaluation of apoptosis using agarose gel electrophoresis (C, FPS-1; F, FMS-1). Only DNA samples from the FMS-1 cells (F) after etodolac treatment for 72 h revealed the DNA ladder, indicating apoptosis. M, marker, 100-bp DNA ladder; E, etodolac treatment; C, control (without etodolac treatment).

Although May-Giemsa staining of FPS-1 cells showed no morphological change, FMS-1 cells showed nuclear fragmentation in the dead cells after 72 h of etodolac treatment (Figure 4A, B, D, E). Agarose gel electrophoresis of extracted DNA samples from the dead cells revealed a DNA ladder, indicating apoptosis in FMS-1 cells (Figure 4F), but not in FPS-1 cells (Figure 4C).

### Activation of Caspase-8, -9, and -3 with Etodolac Treatment

**Figure 5:**
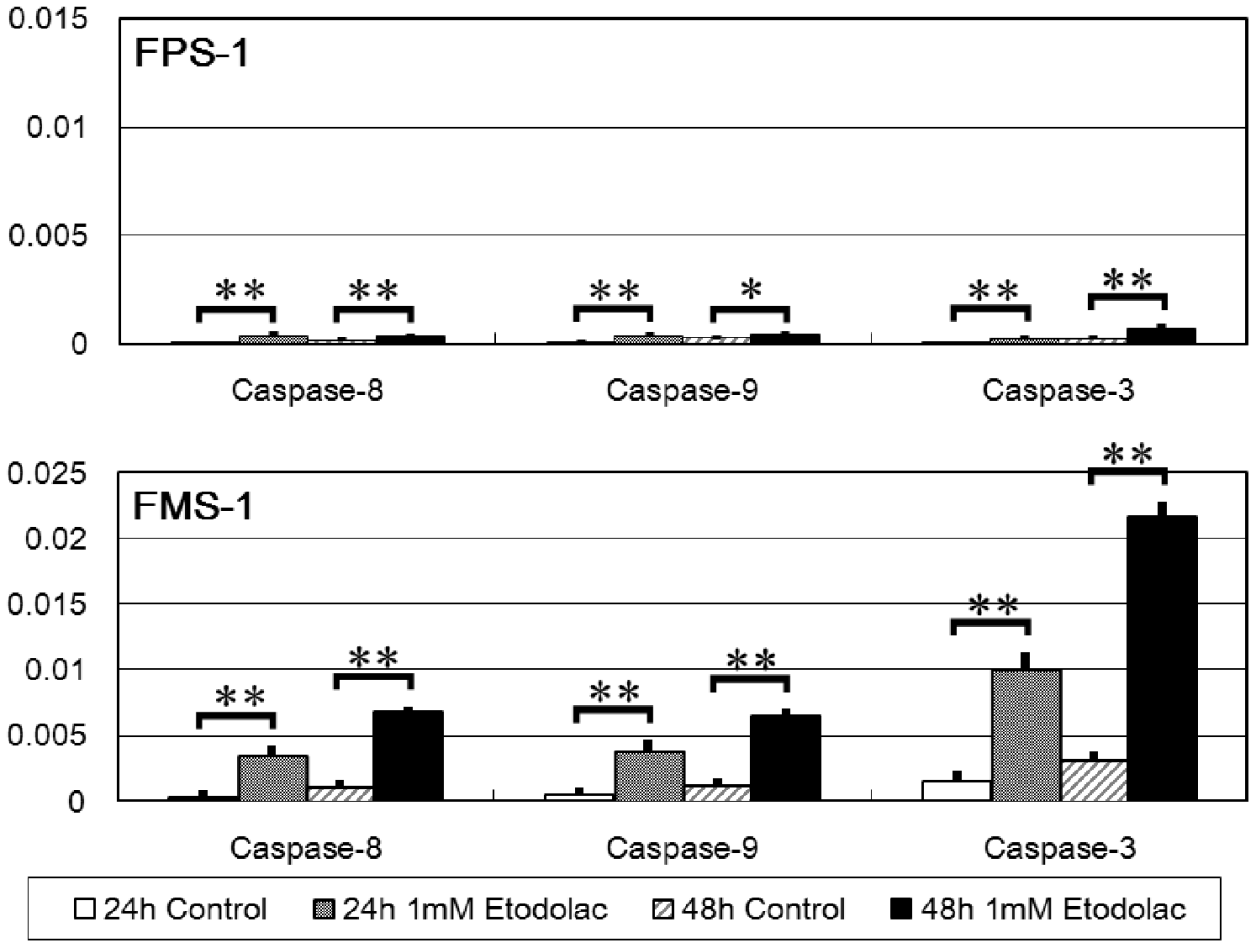
Activities of caspase-8, -9, and -3 with etodolac treatment (24 and 48 h) (n = 3). Both in FPS-1 and FMS-1 cells, etodolac significantly activated caspase-8, -9, and -3 at both 24 and 48 h compared with the control group. However, the differences of caspase-activities were much smaller in FPS-1 cells than those in FMS-1 cells. P-values were calculated based on Student’s t-test (*, P<0.05; **, P<0.01).

Both FPS-1 and FMS-1 cells treated with etodolac showed significant activation of caspase-8, -9, and -3 at 24 or 48 h compared with the control group, which was not treated with etodolac. However, the differences of caspase-activities were much smaller in FPS-1 cells than those in FMS-1 cells (Figure 5).

### Inhibition of Apoptosis by Caspase Inhibitors

**Figure 6:**
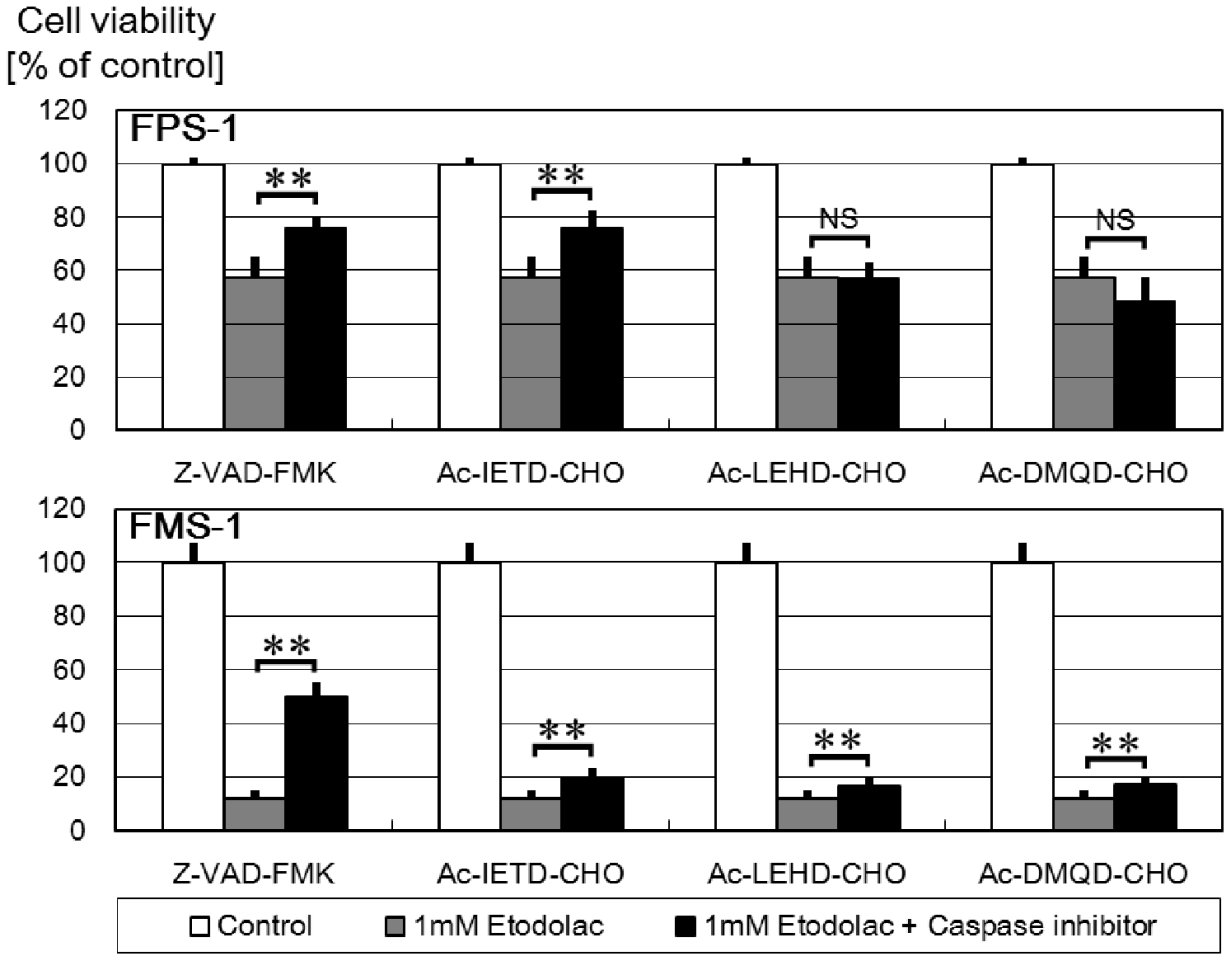
Effects of several caspase inhibitors (Z-VAD-FMK, a broad caspase inhibitor; Ac-LEHD-CHO, a caspase-8 inhibitor; Ac-IETD-CHO, a caspase-9 inhibitor; Ac-DMQD-CHO, a caspase-3 inhibitor) on etodolac treatment (48 h) (n = 5). Each of these caspase inhibitors inhibited etodolac-induced apoptosis of FMS-1 cells incompletely but significantly. On the other hand, in FPS-1 cells, only Z-VAD-FMK and Ac-LEHD-CHO significantly inhibited etodolac-induced apoptosis. P-values of each analysis were calculated based on Student’s t-test (**, P<0.01; NS, not significant).

Each of the caspase inhibitors, Z-VAD-FMK (a broad caspase inhibitor), Ac-LEHD-CHO (a caspase-8 inhibitor), Ac-IETD-CHO (a caspase-9 inhibitor), and Ac-DMQD-CHO (a caspase-3 inhibitor), incompletely but significantly inhibited etodolac-induced apoptosis of FMS-1 cells. Conversely, in FPS-1 cells, only Z-VAD-FMK and Ac-LEHD-CHO significantly inhibited etodolac-induced apoptosis (Figure 6).

## Discussion

The correlation of COX-2 overexpression with prognosis in human bone and soft tissue sarcomas has been reported previously. Although some investigators have affirmed the correlation of COX-2 overexpression with poor prognosis in some sarcomas, including osteosarcoma [7]–[9], chondrosarcoma [6], and uterine leiomyosarcoma [10], other investigators have denied the association in bone and soft tissue sarcomas [24]–[29]. The result of the present study provides the first immunohistochemical evidence that COX-2 overexpression in MPNST is significantly associated with decreased overall survival (P = 0.0495). Moreover, both the univariate and the multivariate analyses revealed that COX-2 overexpression was an independent risk factor for the poor outcome of patients with MPNST. Therefore, the immunohistochemical analysis of COX-2 in MPNST might provide useful prognostic information.

From the review of previous multivariate analyses, we conclude that large tumor size [30], [31], the presence of distant metastasis at the time of diagnosis [31], inadequate surgical treatment (without a wide margin) [30], [32], [33], and not receiving radiation therapy [30], [32] were major unfavorable prognostic factors. In the present study, the multivariate analysis indicated that large tumor size, the presence of distant metastasis at the time of diagnosis, and inadequate surgical treatment were independent risk factors, whereas treatment with radiation therapy did not significantly correlate with poor prognosis. Most of the patients in the present study received radiation therapy as additional treatment after the surgical treatment without a wide margin or as palliative treatment for an inoperable tumor. These radiation therapies did not seem to improve the prognoses in the patients included in the present study.

Though tumor location in the trunk and tumor in the deep tissue are known as risk factors correlated with an unfavorable prognosis in the soft tissue sarcomas [34], the certain association of these factors with an unfavorable outcome in MPNST was controversial in the previous studies employing multivariate analysis; Anghilevi et al. [30] and Endo et al. [35] reported location in the trunk as a poor prognostic factor, whereas Okada et al. [31] and Wong et al. [32] did not show this association; Endo et al. [35] had also reported the association of deep-seated tumors with poor outcome, whereas Anghilevi et al. [30] and Okada et al. [31] did not show this correlation. Likewise, the result of the present study did not show a relationship between the location, including trunk location and depth, and poor overall survival. Therefore, we could not draw a conclusion about the clinical significance of the location either in the trunk or in the deep tissue as a prognostic factor of overall survival in MPNST. Further investigation with a larger number of cases or systematic review of the previous articles will be needed to solve this problem.

There are previously advocated reasons for the correlation of poor prognosis with COX-2 overexpression in many malignant diseases. COX-2 promotes malignancy through direct and indirect mechanisms, that is, prostaglandins may directly stimulate mitogenesis through a direct effect on fibroblasts, while COX-2 indirectly affects mutagenesis, angiogenesis, increased cell migration, and apoptosis, and it even affects the activity of cells with cytotoxic function, all which regulate the ability of malignant cells to survive [36], [37]. Thus, elevated COX-2 expression is often correlated with decreased survival of patients with malignancies. The results of the present study indicate that the avoidance of apoptosis seems to be related to survival and the progression in FMS-1, and this may lead to poor overall survival in patients with MPNST.

The present study also revealed that etodolac, a selective COX-2 inhibitor, induced cell death in the MPNST cell line, FMS-1, and this death was demonstrated to be apoptotic by the results of a morphological examination (apoptotic body), agarose gel electrophoresis (DNA ladder), and evaluation of caspase activation and efficacy of caspase-blockage. Usually, caspase-dependent apoptosis occurs through two main interconnected pathways, which are intrinsic and extrinsic pathways. The intrinsic pathway or intracellular path is mediated by the Bcl-2 family, whereas the death receptor or extrinsic pathway is activated by signals from other cells [38]. In the intrinsic pathway, progression through the pathway usually leads to activation of caspase-9. On the other hand, activation of caspase-8 occurs in the extrinsic pathway. The activated caspase-9 or -8 then cleaves procaspase-3, giving activated caspase-3, which acts as an executioner of apoptosis.

The apoptotic event was shown to occur in FMS-1 cells through the activation of caspase-8, -9, and -3, as these caspases were activated in the FMS-1 cells treated with etodolac. Additionally, etodolac-induced apoptosis was inhibited by each caspase inhibitor, broad caspase inhibitor Z-VAD-FMK, caspase-8 inhibitor Ac-IETD-CHO, caspase-9 inhibitor Ac-LEHD-CHO, and caspase-3 inhibitor Ac-DMQD-CHO. Therefore, the results of the present study suggest that the caspase cascade of etodolac-induced apoptosis of FMS-1 cells may involve both intrinsic and extrinsic pathways. The results of the present study also suggest that there are other pathways independent of caspase activation, because the efficacy of the broad caspase inhibitor Z-VAD-FMK was incomplete. Although the UPS cell line, FPS-1, also showed COX-2 overexpression [22], the efficacy of etodolac was different from that in FMS-1. There have been several reports on the molecular mechanisms or factors involved in selective COX-2 inhibitor-induced apoptosis; down-regulation in bcl-2 protein expression [39], augmentation of TRAIL (tumor necrosis factor-related apoptosis-inducing ligand)-induced apoptosis [40], augmentation of Fas-mediated apoptosis [41], activation of the cytochrome c-dependent pathway [42], blocking of Akt activation [43], and increase in arachidonic acid and ceramide [44]. In addition, apoptotic pathways in the selective COX-2 inhibitor-induced apoptosis of tumor cells depend on tumor cell types or selective COX-2 inhibitor types. However, the signaling pathways from the first signal initiated by etodolac to caspase activation have not been sufficiently elucidated. Therefore, it is necessary to investigate how caspase activation occurs in FMS-1 cells and to determine whether there are other pathways independent from caspase activation, as well as to evaluate the anti-tumor effect of selective COX-2 inhibitors in other MPNST cell lines in future research.

Clinically, the antitumor effect of NSAIDs, including selective COX-2 inhibitors, for the treatment or prevention of some carcinomas has been reported [45], and the efficacy of the combination therapy with a selective COX-2 inhibitor and other chemotherapeutic agents has been reported in advanced sarcomas [46]. Therefore, the prevention of carcinogenesis of MPNST in the patients with NF1, like a colorectal cancer in the patients with familial adenomatous polyposis [45], the prevention of tumor recurrence after the surgical treatment for MPNST, as well as the therapeutic effect by induction of apoptosis in the treatment of MPNST should be evaluated in future clinical investigation.

Etodolac is one of the most common selective COX-2 inhibitor and widely used all over the world for a long time (approximately 20 years). Though the risk of cardiovascular events caused by the side effect of selective COX-2 inhibitors is widely known, the risk of normal clinical dose of etodolac is still obscure because of the limited number of previous studies [47]. Thus, when we clinically use etodolac for the treatment of malignancies, we must deliberate about the risk and benefit.

Although 1 mM of etodolac (287.4 µg/ml) showed marked anti-tumor effect for MPNST in the present study, Cmax of the usual dose of etodolac was reported as 15.9 µg/ml (200 mg, orally) and 34.0 µg/ml (400 mg, orally) [48], much lower than the concentration used in this study. This is the limitation of this study. However, 0.125 mM of etodolac (35.9 µg/ml) also inhibited cell growth of FMS-1 *in vitro* (Fig. 3). Further investigation with lower concentration of etodolac (e.g. 0.125 mM) will be needed.

In conclusion, we analyzed the relationship between COX-2 overexpression and prognosis in patients with MPNST. Overexpression (>50% positive cells) of COX-2 was significantly associated with poor prognosis in these patients. Moreover, etodolac, a selective COX-2 inhibitor, induced apoptosis of FMS-1 cells through the activation of caspase-8, -9, and -3. Although the selective COX-2 inhibitor-induced apoptosis of some sarcoma cells has been reported previously, the present report is the first, to our knowledge, to cover the apoptosis of MPNST cells induced by the selective COX-2 inhibitor etodolac. Selective COX-2 inhibitors, including etodolac, are in widespread use as NSAIDs against inflammatory disease. The results of this study may reveal a therapeutic hypothesis in the context of a molecular chemotherapeutic approach to treating MPNST.

## Materials and Methods

### Tumor Samples

Forty-four Japanese patients with primary high-grade MPNST treated at university hospitals belonging to the “Tohoku Musculoskeletal Tumor Society” between 1992 and 2008 were included in this study. The histologic diagnosis was based on the criteria for diagnosis of MPNST previously outlined by Fletcher [49], [50].

All the patients had been treated at our hospitals and followed up at our clinics. Clinical details and follow-up information were obtained by reviewing the patients’ medical charts. The patients comprised 21 males and 23 females between the ages of 15 and 86 years (median, 52 years; mean, 49.8 years) and included 12 patients with familial NF1 and 9 patients with solitary NF1. The clinical diagnosis of NF1 was based on the NIH criteria [51]. In 24 cases, a relationship to the peripheral nerve was found, whereas no such relationship was seen in 20 cases. The tumors were located in the trunk in 20 cases and in the extremities in 24 cases. Twelve tumors were located superficially, 31 were deep-seated, and 1 case was uncertain. The largest diameters of the tumors ranged from 2 to 39 cm (median, 8.7 cm; mean, 10.8 cm). Distant metastasis was found in 11 cases at the time of diagnosis. The surgical procedures used to treat the patients, excluding the patients without surgical treatment (biopsy only), were classified by the margin as wide resection or not wide resection (including intralesional and marginal) according to the definition of Enneking and Maale [52]. Seven cases (15.9%) received a biopsy only, while 24 (54.5%) and 13 (29.5%) cases received wide or not wide resection, respectively. Wide resection was considered to be curative surgical treatment, whereas not wide resection and biopsy only were considered to be not curative treatment. Additionally, 11 out of 44 (25.0%) patients received chemotherapy, whereas 14 out of 44 (31.8%) patients received radiation therapy.

The follow-up period was dated from the time of diagnosis, the median follow-up period was 24 months, and the mean follow-up period was 37 months. Overall survival was recorded up until the time of death.

### Immunohistochemical Analysis of COX-2 Expression in Tumor Samples

All the specimens used in the present study were resected before the preoperative chemo- and/or radiotherapy. Immunohistochemical analysis was performed on the tissue sections from paraffin-embedded blocks using the streptavidin-biotin complex (SABC) method. The mouse monoclonal anti-COX-2 antibody (clone 4H12; diluted 1∶100; Novocastra, Newcastle upon Tyne, UK) was used in this study.

The immunohistochemical results were evaluated by two investigators (H.H, an expert pathologist, and MH) who were unaware of the clinical status of the patients. The sections were examined using a multi-head microscope, and a consensus judgment was adopted as the proper immunohistochemical score of the tumor based on the proportion of stained tumor cells, as follows: 0, no stained tumor cells; 1+, less than 10% of all tumor cells stained; 2+, 10–50% of all tumor cells stained; 3+, 50–90% of all tumor cells stained; 4+, more than 90% of all tumor cells stained. Immunohistochemical scores of 3+ and 4+ (≥50% positive cells) were regarded as indicating the overexpression of COX-2.

### Cell Line and Reagents

The human MPNST cell line FMS-1 [23] and the human UPS cell line FPS-1 [22] used in the present study, were established in our laboratory., Both FMS-1 and FPS-1 cells showed overexpression of COX-2, as demonstrated by immunohistochemistry, Western blotting, and reverse transcription-polymerase chain reaction analysis [51], [52]. Cells were grown in RPMI-1640 medium (Sigma R8758, St. Louis, MO, USA) supplemented with 15% heat-inactivated fetal calf serum (FCS) (JRH Biosciences, Lenexa, KS, USA), 50 units/ml penicillin G, and 50 µg/ml streptomycin. The cells were inoculated into 25-cm^2^ tissue culture flasks (Iwaki Glass, Tokyo, Japan) and incubated at 37°C in a humidified atmosphere with 5% CO_2_.

Etodolac, a selective COX-2 inhibitor, was provided by Nippon Shinyaku Co. (Kyoto, Japan). A stock solution of etodolac was prepared in 99.5% ethanol and stored at −80°C. Caspase inhibitors (Z-VAD-FMK, broad caspase inhibitor; Ac-IETD-CHO, caspase-8 inhibitor; Ac-LEHD-CHO, caspase-9 inhibitor; Ac-DMQD-CHO, caspase-3 inhibitor) were obtained from Peptide Institute, Inc. (Osaka, Japan) and were dissolved in dimethyl sulfoxide (DMSO) as stock solutions.

### Measurement of Etodolac-induced Cell Death *in vitro*

Cell viability was assessed using a cell proliferation reagent, WST-1 (Roche Diagnostics, Mannheim, Germany) [53]. FMS-1 and FPS-1 cells were seeded in 96-well plates (BD Falcon 353072, BD Biosciences, Franklin Lakes, NJ, USA) at a dose of 1.0×10^4^ cells/well and incubated for 24 h at 37°C in 100 µl of phenol-red-free RPMI-1640 medium (Sigma R7509) supplemented with 10% FCS, 50 units/ml penicillin G, 50 µg/ml streptomycin, and 200 mM L-glutamine. FMS-1 and FPS-1 cells were treated by adding etodolac and then were dissolved and diluted to the desired concentration by 1 µl of 99.5% ethanol with 49 µl of culture medium (final concentration of ethanol: 0.67% or less), at final concentrations of 0 (control), 0.125, 0.25, 0.5, and 1 mM. At 24, 48, and 72 h after etodolac treatment, 15 µl of WST-1 reagent was added to each well, and the plates were incubated for 2 h at 37°C. The plates were then shaken for 1 min at room temperature, and the absorbance of 450–655 nm was measured using a multi-well ELISA reader (Microplate Reader, Model 550, Bio-Rad Laboratories, Hercules, CA, USA).

### Morphological Analysis and DNA Fragmentation Analysis of Dead Cells

FMS-1 and FPS-1 cells were seeded in 6-well plates (BD Falcon 353046) at a dose of 5.0×10^5^ cells/well and incubated for 24 h at 37°C in 5 ml of culture medium. Cells were treated by adding etodolac, dissolved and diluted to the desired concentration by 50 µl of 99.5% ethanol (final concentration of ethanol: 0.99% or less), at a final concentration of 0 (control) or 1 mM. At 24, 48, and 72 h after the etodolac treatment, cells were removed using a rubber policeman. For morphological analysis of dead cells, 50 µl of the cell suspension was cytospun on silane-coated slides, which were stained by May-Giemsa staining and observed by two investigators (HH and MH). Suspended cells were washed in ice-cold phosphate-buffered saline (PBS) twice and pelleted by centrifugation. To detect cell death, whether apoptotic or not, we extracted a DNA sample from each cell pellet using the Enhanced Apoptotic DNA Ladder Detection Kit (BioVision, Mountain View, CA, USA) according to the manufacturer’s protocol. Extracted DNA samples were electrophoresed through 1.8% agarose gels and stained with ethidium bromide.

### Measurement of Caspase-8, -9, and -3 Activity

FMS-1 and FPS-1 cells were seeded in 6-well plates at a dose of 5.0×10^5^ cells/well and incubated for 24 h at 37°C in 5 ml of culture medium. FMS-1 cells were treated by adding 0 (control) or 1 mM etodolac, dissolved, and diluted to the desired concentration by 50 µl of 99.5% ethanol (final concentration of ethanol: 0.99% or less). At 24 and 48 h after etodolac treatment, cells were removed with a rubber policeman. Suspended cells were washed in ice-cold PBS twice and pelleted by centrifugation. To detect the activity of caspase-8, -9, or -3, we extracted samples from cell pellets and measured them using the APOPCYTO Caspase-8, -9, or -3 Colorimetric Assay Kit (MBL, Nagoya, Japan), according to the manufacturer’s protocol. The 96-well plates were then shaken for 1 min at room temperature, and absorbance at 405 nm was measured using a multi-well ELISA reader. The activities of the caspases were corrected by protein concentrations.

### Blockage of Apoptosis by Various Caspase Inhibitors

Cell viability was assessed by the WST-1 method. FMS-1 and FPS-1 cells were seeded in 96-well plates at a dose of 1.0×10^4^ cells/well and incubated for 24 h at 37°C in 100 µl of phenol-red-free medium. After 2 hours of preincubation with 0 (control) or 100 µM caspase inhibitors (Z-VAD-FMK, Ac-IETD-CHO, Ac-LEHD-CHO, or Ac-DMQD-CHO), dissolved and diluted to the desired concentration by 0.75 µl of DMSO with 39.25 µl of culture medium (final concentration of DMSO: 0.5% or less), Cells were treated by adding etodolac, dissolved, and diluted to the desired concentration by 1.5 µl of 99.5% ethanol with 8.5 µl of culture medium (final concentration of ethanol: 1% or less), at a final concentration of 0 (control) or 1 mM. At 24 and 48 h after etodolac treatment, 15 µl of the WST-1 reagent was added to each well and the plates were incubated for 2 h at 37°C. The plates were then shaken for 1 min at room temperature, and the absorbance of 450–655 nm was measured using a multi-well ELISA reader.

### Data Analysis

In the clinicopathological analysis, Pearson’s chi-square test was used to examine correlations between two dichotomous variables. The overall survival curves were calculated using the Kaplan-Meier method, and the differences were compared by the log-rank test. The Cox proportional hazards model was carried out to estimate the hazard ratios for positive risk factors for death.

In the *in vitro* examinations, all data were summarized as the means ± SD. Statistical analysis was performed using an unpaired Student’s t-test.

In all examinations, P<0.05 was considered statistically significant. The data analysis was performed using the JMP 9.0 statistical software package (SAS Institute, Cary, NC, USA).

### Ethics Statement

This study was approved by the ethical review committee of Fukushima Medical University (No. 1076). Written informed consent for study inclusion was obtained from all patients and/or their families.
